# Detection of Human Neutrophil Elastase by Fluorescent Peptide Sensors Conjugated to TEMPO-Oxidized Nanofibrillated Cellulose

**DOI:** 10.3390/ijms23063101

**Published:** 2022-03-13

**Authors:** Robert T. Mackin, Krystal R. Fontenot, Judson Vincent Edwards, Nicolette T. Prevost, Jacobs H. Jordan, Michael W. Easson, Brian D. Condon, Alfred D. French

**Affiliations:** United States Department of Agriculture, Agriculture Research Service, Southern Regional Research Center (USDA-ARS-SRRC), New Orleans, LA 70124, USA; rmackin87@gmail.com (R.T.M.); kfont22@gmail.com (K.R.F.); nicolette.prevost@usda.gov (N.T.P.); jacobs.jordan@usda.gov (J.H.J.); michael.easson@usda.gov (M.W.E.); brian.condon@usda.gov (B.D.C.); al.french@usda.gov (A.D.F.)

**Keywords:** biosensors, TEMPO-oxidized nanofibrillated cellulose, human neutrophil elastase, peptides, computational modeling

## Abstract

Peptide–cellulose conjugates designed for use as optical protease sensors have gained interest for point-of-care (POC) detection. Elevated serine protease levels are often found in patients with chronic illnesses, necessitating optimal biosensor design for POC assessment. Nanocellulose provides a platform for protease sensors as a transducer surface, and the employment of nanocellulose in this capacity combines its biocompatibility and high specific surface area properties to confer sensitive detection of dilute biomarkers. However, a basic understanding of the spatiotemporal relationships of the transducer surface and sensor disposition is needed to improve protease sensor design and development. Here, we examine a tripeptide, fluorogenic elastase biosensor attached to TEMPO-oxidized nanofibrillated cellulose via a polyethylene glycol linker. The synthetic conjugate was found to be active in the presence of human neutrophil elastase at levels comparable to other cellulose-based biosensors. Computational models examined the relationship of the sensor molecule to the transducer surface. The results illustrate differences in two crystallite transducer surfaces ((110) vs. (1−10)) and reveal preferred orientations of the sensor. Finally, a determination of the relative (110) vs. (1−10) orientations of crystals extracted from cotton demonstrates a preference for the (1−10) conformer. This model study potentiates the HNE sensor results for enhanced sensor activity design.

## 1. Introduction

TEMPO-oxidized nanofibrillated cellulose (tNFC) has received increased attention in recent years as a highly functional hydrogel for tissue engineering and wound healing applications [1,2,3]. The properties conferred by the hydrogel’s high specific surface area and hydrophilic nanofibril network promote adoption of a three-dimensional matrix that allows mimicry of a biological environment as is found in the extracellular matrix (ECM), the body’s scaffold for cells in tissues and organs [4,5]. The structure of cellulose derivatives such as cellulose nanocrystals (CNC) and tNFC can provide a valuable platform for in vitro and in vivo sensor design and application through their highly tunable nature and biocompatibility [6,7], and work on combining TEMPO-oxidized CNC and ECM has become a field of increasing interest [8,9]. For instance, recent work by Powell et al. has proven that tNFC-based dressings inhibit the growth of P. aeruginosa, which are often prevalent in chronic wounds [10,11]. Notably, when contrasted as a biocompatible hydrogel with other forms of nanocellulose, both CNC and tNFC facilitate interfacial binding of biomolecules [12,13,14]. For example, CNC affords a platform for biosensor design where a high specific surface area required for effective sensor transducer surfaces promotes increased sensor sensitivity as a function of crystallite size and disposition of the sensor molecule [15].

Recent work into point-of-care (POC) diagnostics has focused on applications related to chronic illnesses, examining advances and limitations of current technologies [16,17,18], and studies utilizing cellulose crystallites as the basis for the biosensing device have explored their use on chronic wounds specifically [19,20]. While a complete biosensor (and POC diagnostic) requires an integrated signal output, these studies utilizing cellulose crystallites as a transducer surface and anchoring substrate for the bioactive molecule offer the foundation for developing fully functional biosensor systems. As such, numerous nanocellulose-based sensor motifs have been shown to be especially effective in measuring protease levels in chronic wound fluid and to retain activity interfaced with protease modulating dressing designs [21]. Integration of these cellulose-based biosensing systems into wound dressings provides the basis for a novel POC diagnostic device, making it possible to identify markers of chronic wounds and granting earlier detection and diagnosis of major health issues for the patient. However, as observed structurally, CNC provides a 2D sensor anchor—a flat rigid surface—for bio-sensing models in wound healing, whereas tNFC, which has also been targeted for in situ detection and real time in vivo monitoring, is composed of a 3D structure [22,23,24,25].

It is important to consider the effect of substrate, linker, and anchor on sensor sensitivity from a perspective of enzyme recognition properties [26,27]. Sensor recognition in the context of enzyme substrate kinetics is a function of the analyte turnover rate (k_cat_) and binding affinity (K_m_). In nanocellulose-based approaches developed for protease detection, the sensor component has been designed as an enzyme substrate immobilized to the transducer surface, and the enzyme functions as the analyte [27,28].

The efficacy of a sensor when placed in a physiological environment is dependent on the biomaterial’s hydration energy and spatiotemporal properties, which influence molecular recognition and biocompatibility [29,30]. Hydrogel porosity, material formability, and strength play an important role in designing in vivo activated sensors grafted in situ on an active organ [31] or on bones for detection of osteoporosis [32]. Many of these properties are tunable via chemical parameters such as degree of methylation or by external forces including heat and pH [33,34]. Similarly, tNFC is a hydrogel adaptable to the natural cellular environment of tissues and organs with uniform incorporation into the ECM [8,9]. Recently, cellulosic hydrogels and aerogels were reported as sensor transducer surfaces with this observation in mind [27,35]. Moreover, when employed both singularly and in combination with other biopolymers, tNFC facilitates in situ performance of sensors [36].

Here, we investigate the ability of tNFC with conjugated enzyme substrate and fluorophore as a biosensing unit to detect human neutrophil elastase (HNE), a serine protease often found at elevated concentrations in chronic wounds. The results of this study are compared to results from materials measured previously [20,27]. Additionally, we outline an approach to evaluating the potential for the bioactive system to detect HNE based on the inter-related influences of sensor, linker, and anchor as determined by a computational model developed for tNFC constructed from a single layer of (110) and (1−10) TEMPO-oxidized cellulose crystal planes. Finally, we examine related cellulose-based materials building on the computation findings and assess their potential to be utilized as a peptide–cellulose conjugate for biosensing applications. Thus, the results of this study open the way for novel peptide–cellulose conjugate material designs as the foundation for POC diagnostics leading to diagnostic devices with increased sensitivity.

## 2. Results and Discussion

### 2.1. Surface Morphology

**Figure 1:**
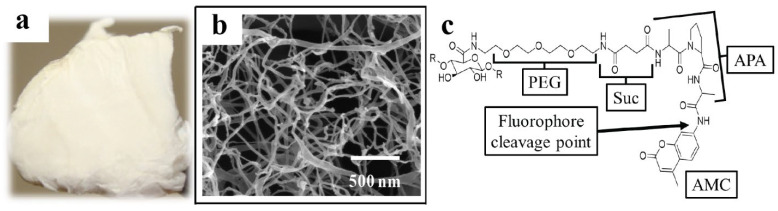
(**a**) Optical and (**b**) FE-SEM images of tNFC. The FE-SEM images were produced at 80,000× magnification with a 500 nm scale, and (**c**) the major components of the linker–fluorogenic elastase substrate, which is composed of PEG linker, Suc-APA elastase substrate, and AMC fluorophore. The AMC cleavage point is highlighted by the arrow.

Figure 1 presents the optical and field-emission scanning electron microscopy (FE-SEM) images of the tNFC sample. As can be seen in the optical microscopy image (Figure 1a), the tNFC material adopts a robust yet lightweight 3D scaffold that can be utilized as the basis for ECM. However, the SEM image (Figure 1b) illustrates the incredibly low density and high degree of porosity of the material. The image presents a nanofibrillar network composed of cellulose fibrils up to tens of nanometers in width but microns in length, which is comparable to that of nanocellulosic aerogel (NA), [35] and shows the presence of both mesopores and macropores. Attached to the TEMPO-oxidized cellulose surface is the bioactive molecule that serves as the basis for the biosensor, the molecular structure of which is provided in Figure 1c, with a breakdown of the important structural segments. Of note are the polyethylene glycol (PEG) linker, which aids in extending the sensor away from the transducer surface, the tripeptide composed of a succinate (Suc) connector and Ala-Pro-Ala (APA), and the scissile bond (fluorophore cleavage point) connecting the alanine of the tripeptide to the coumarin fluorophore. This bond is broken when the biosensor or bioactive molecule interacts with HNE, releasing the 7-amino-4-methylcoumarin (AMC), which is then detected via fluorescence spectroscopy.

### 2.2. Bioactivity Assays with HNE

**Figure 2:**
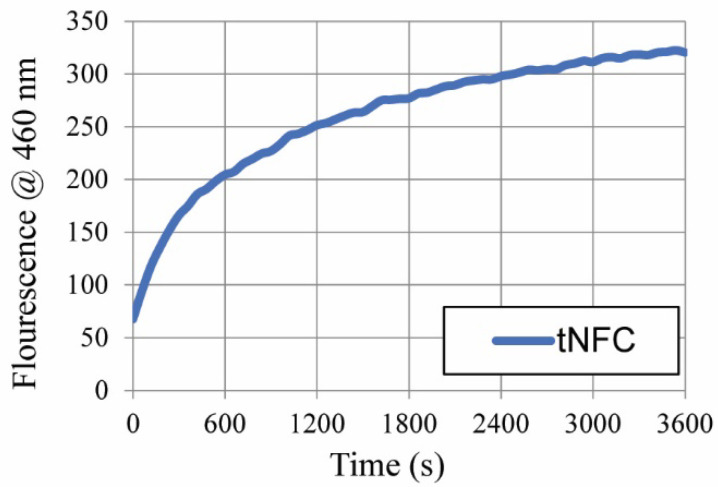
Response curves of 2 mg of tNFC biosensors upon detection of 7-amino-4-methylcoumarin released with HNE at 0.5 U/mL substrate hydrolysis at 37 °C.

The ability of the system to detect HNE activity is assessed by applying the enzyme to the bioactive molecule analogous to a POC diagnostic having the interrogated sample placed onto it [37,38,39]. However, the system in this study is not a complete biosensor, as it lacks integrated signal output and requires an external fluorescence measurement currently. Although, it provides the foundation for complete POC diagnostic design as it is further optimized and developed. A comparison of two-dimensional (2D) analogs provides a perspective analysis of the properties that may be provided by a biocompatible in vivo sensor. Similar studies using AMC to assess the bioactive response of the HNE enzyme substrate illustrate the functional role of the fluorophore as a sensitive signal alternative to colorimetric detection [15,20,27]. In contrast to the previous studies where a glycine linker was utilized, here the tripeptide elastase substrate is attached to the transducer surface via a PEG linker to probe the structure function relations between the sensor and transducer surface. To assess the system sensitivity, the fluorescence is measured over time as a function of the protease-catalyzed release of the COOH-terminal AMC unit. The results from the HNE reaction progress curve for tNFC-Pep are provided in Figure 2. The fluorescence response curve with 2 mg of the tNFC-Pep biosensor demonstrates enzyme activity assessed at 0.5 U HNE/mL.

The fluorescence progress curve (Figure 2) peaks at approximately 320 fluorescence units at the 60 min time point. Previous studies of peptide–cellulose conjugates have shown similar HNE reactivity profiles [20,27] for a variety of cellulose-based materials, including wood-based cellulose nanocrystals (wCNC) and nanocellulose composites (wNCC), cotton nanocellulosic aerogel (NA), and cotton-based print cloth (PC) fabric and filter paper (FP), the results of which are presented in Table 1. Note, the NCC samples are composed of a mixture of nano- and microcrystalline cellulose, which are denoted by the ratio after each entry. When compared with analogs employing 2D nanocellulose transducer surfaces (CNC, NCC, PC, and FP), tNFC-Pep exceeds previously observed values determined for cellulose–peptide conjugates measured at similar enzyme levels. Notably, the tNFC-Pep analog differs from 2D analogs by way of the extended PEG linker. In addition to the fluorescence values, the corresponding crystallite volumes and number of sensors per volume for each material are provided in Table 1 for comparison. These values were previously determined based on the crystallite models, illustrating the average number of cellulose residues for each material, and the degree of substitution of the biosensors, providing the number of anchored bioactive molecules per glucose residue [40]. Note, the 2D materials were measured at 0.05 U/mL, while the bioactivity measurements for tNFC-Pep and NA-Pep, which share a high degree of porosity and adopt a 3D network, were both assessed at an enzyme concentration of 0.06 U/mL. Notably, a linear scaling factor was applied to normalize the fluorescent results of these analogs with the 2D materials for comparison. 

The presence and activity of human neutrophil elastase (HNE) in chronic wounds can greatly hinder wound healing by degrading the body’s necessary growth factors. In chronic wounds, it was previously shown that HNE at a concentration of 1 mU/mg protein was enough to obstruct repair and regeneration in the wound [41]. This same study reported elastase activity in both pressure and leg ulcers ranging from 1–50 milliunits (U/mL), which is much higher than the typical amount of HNE found in healing wounds (0.1 U/mL) [41]. Previous work on peptide–cellulose biosensors illustrated that the sensitivity of the sensor was enough to adequately detect levels of HNE found in chronic wounds (0.015 U/mL) [20].

### 2.3. Assessment of the Influence of Volume Ratio on Sensor Activity

**Figure 3:**
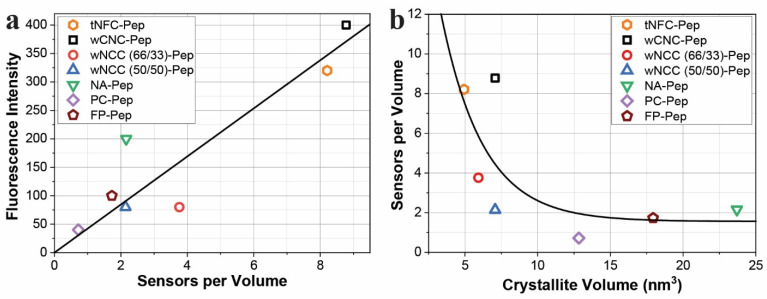
(**a**) Fluorescence intensity based on the number of sensors per crystallite volume, and (**b**) sensors per crystallite volume as a function of the calculated crystallite volume.

We created a visualization of sensor functionality through a 3D model that depicts the relative contribution of sensor volume occupied on the transducer, the TEMPO-oxidized cellulose surface. This is considered in light of binding of HNE and transmission of the fluorescent signal. The model of the sensor volume component was created based on a range of crystallite determinations to illustrate the effectiveness of the 3D component (PEG–elastase substrate) in tNFC-Pep. The number of sensors per unit volume of crystallite has been employed to delineate the detection efficiency of nanocellulose as a transducer surface. This is illustrated in Figure 3a, which presents the fluorescence intensity of cellulose-based HNE sensors as a function of the number of biosensors per volume. The tNFC-Pep material produces one of the most intense fluorescence signals at 0.5 U/mL enzyme concentration and contains one of the largest amounts of sensors per volume, suggesting that a greater number of bioactive molecules provides a stronger spectral intensity. Overall, the trend of increasing sensors per volume resulting in an increasing fluorescence intensity appears linear as is illustrated with the fit line (Figure 3a, black line). However, an exception is noted in the case of composite materials (wNCC), which contain a mid-range number of sensors per volume but yield a fluorescence intensity lower than expected. This is attributed to crystal aggregation during composite formation limiting HNE access to the enzyme substrate [21] and appears to decrease with an increasing nanocrystalline component. Conversely, the nanocellulosic aerogel (NA) contains a similar number of sensors per volume as the wNCC (50/50) sample yet produces a markedly higher fluorescence intensity, a factor of the hydrogel-like nature of the NA material, which swells considerably. The retention of liquid provides increased surface area, which can increase accessibility of the HNE to the enzyme substrate.

Figure 3b expands on this analysis and shows that as the crystallite size decreases, the number of sensors per unit volume increases and produces a stronger fluorescence intensity as supported by tNFC-Pep (hexagon) and wCNC-Pep (square). This trend is generally consistent with the rank order of sensor volume: wCNC > tNFC > wNCC (66/33) > NA~wNCC (50/50) > FP > PC. Moreover, the results suggest that developing smaller cellulose crystallites could allow for a larger number of bioactive molecules per volume (assuming comparable levels of DS), leading to stronger spectral intensity and a more sensitive detection system based on the corresponding exponential fit line provided in the figure.

### 2.4. Computational Modeling

Previous computational studies on cellulose have examined the properties of smaller structures such as cellobiose and cellotriose and their derivatives, many of which focus on crystal packing and cellulose type (I, II, amorphous, etc.) [42,43,44,45,46,47,48]. While researchers have previously modeled TEMPO-oxidized cellulose fibrils [49], extensive computational models of TEMPO-oxidized cellulose crystallite surfaces focusing specifically on the interactions with attached biomolecules have not been addressed in detail. In this study, computational modeling was performed to assess the spatiotemporal relationship between the transducer surface and biosensing unit and to provide insight into the localized structures.

**Figure 4:**
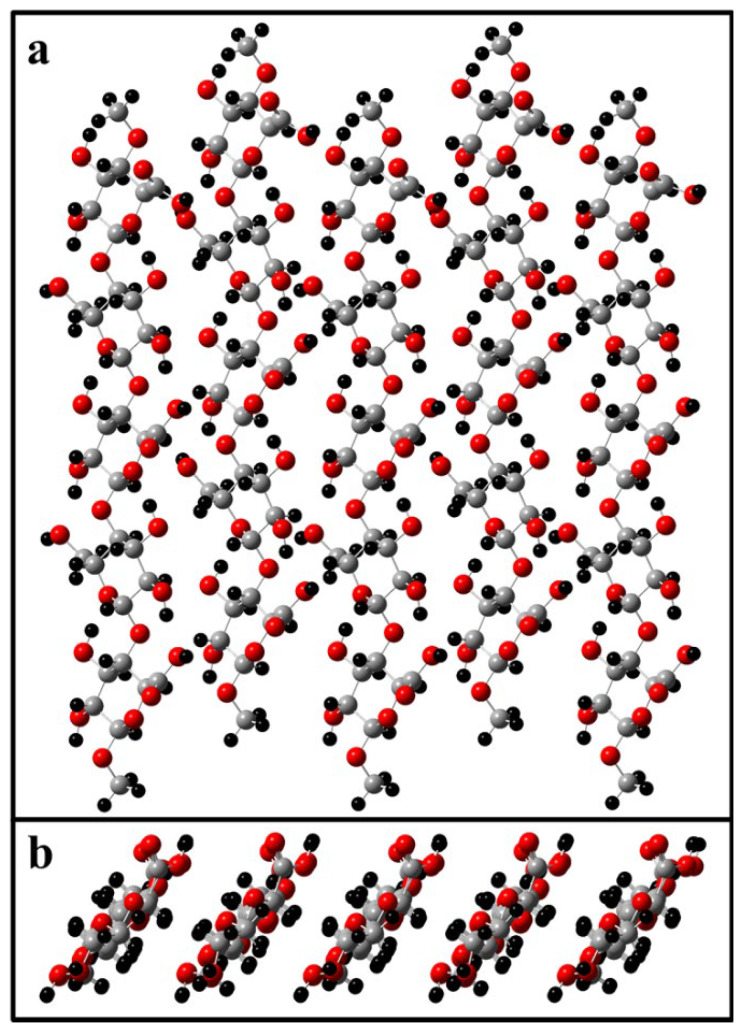
(**a**) Top-down view and (**b**) side view of the 5 × 5 TEMPO-oxidized transducer surface ((1−10) crystal plane) model without attached biosensor. In this model, the grey atoms correspond to carbon, red is for oxygen, and black for hydrogen.

Energy minimization calculations were performed on two TEMPO-oxidized cellulose surfaces, (1−10) and (110) crystal planes, using the semi-empirical (PM3) method (See methods for full computational details). In short, the cellulose surfaces were simulated via five adjacent cellopentaose strands. The C6-hydroxyl was converted to a carboxylic acid for each C6 on the transducer surface, resulting in an alternating pattern of oxidized rings due to the inherent two-fold screw axis of cellulose chains [50]. Figure 4 presents the structure of the TEMPO-oxidized (1−10) crystal plane used in this modeling. The PEGylated linker was attached to the C6 of the central glucan ring, and the attachment conformation was modified for each iterative calculation.

**Figure 5:**
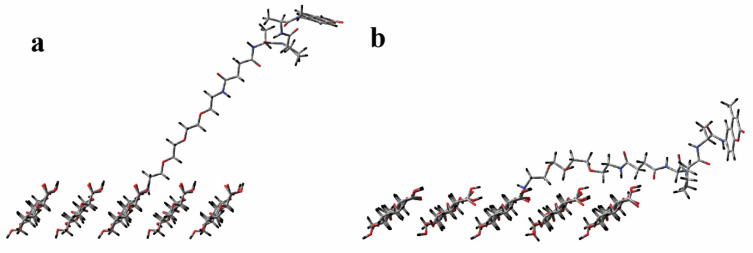
Side view of the optimized peptide–cellulose conjugates (tNFC-Pep) for the (**a**) (1−10) crystal plane and the (**b**) (110) crystal plane.

The two surfaces are shown with the attached biosensor unit in Figure 4 where the structure is shown in the optimal (lowest energy) configuration for both. For the (1−10) crystal plane (Figure 5a), the biosensor emerges from the transducer surface and continues in an upward trajectory, allowing ample space for the HNE to interact with the enzyme substrate and to cleave the AMC unit. However, the PEG linker attachment to the (110) crystal plane (Figure 5b) lies much closer to the transducer surface. As a result, the AMC cleavage point remains generally less accessible to the much larger HNE structure.

**Figure 6:**
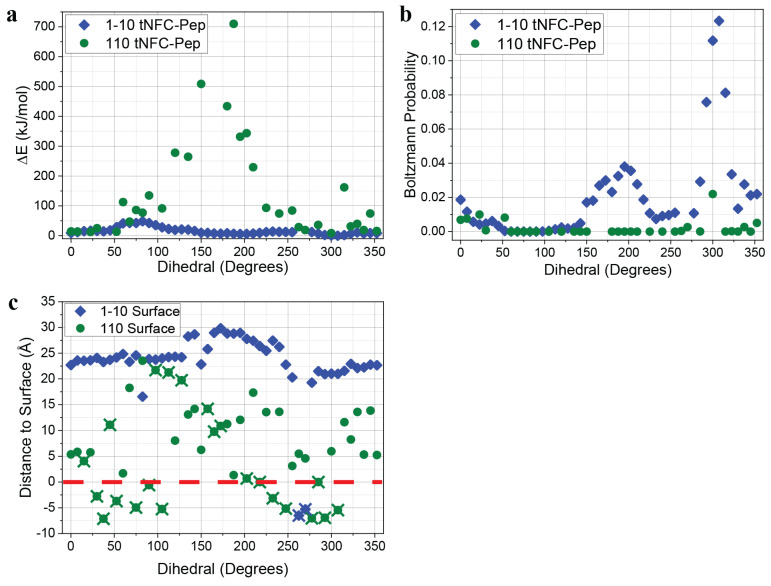
(**a**) Minimized energy for each crystal surface with attached biosensor unit (tNFC-Pep) as a function of the dihedral angle, (**b**) Boltzmann distributions for each surface based on their optimized energies, and (**c**) the distance from the AMC cleavage point to the transducer surface (red dashed line) based on the optimized geometries for each crystal surface. The symbols with an “X” indicate geometries, which did not converge. Thus, the symbols with an “X” indicate calculations that did not fully converge, often because the initial geometry resulted in atomic overlap and are not considered viable structures.

The results and analysis of the energy minimization for each surface as a function of the C2-C5-C6-O_TO_ dihedral angle are shown in Figure 6, where the carbon atoms are part of the same glucan ring, and the oxygen is connected to C6 via a double bond. At zero degrees, the carbonyl group is pointing away from and perpendicular to the transducer surface. Although there are strong differences between the minimized energies of the surfaces, both (110) and (1−10) surfaces have optimized energies around the 300° dihedral. As seen in the figure (Figure 6a), changing the dihedral angle can drastically shift the energy. For the tNFC-Pep (1−10) crystal surface (Figure 6a, blue diamonds), the overall energy (in kJ/mol) of the structure only changes by less than 50 kJ/mol across the entire dihedral range. The calculations reach a maximum around 75° due to the proximity of the N-H group anchored to the transducer surface and a hydroxyl group on an adjacent glucan ring. Otherwise, the optimized energies are similar for every conformation. In contrast, the energy of the (110)-transducer surface (Figure 6a, green circles) is highly dependent upon the orientation of the PEG–Pep unit, with a substantial increase in the energy of the model within the 100–250° range. This orientation results in the proximity of the biosensor to the transducer surface as illustrated in Figure 5. When the rigid structure of the linker forces the biosensor to lie across the surface, the steric interactions cause a sharp increase in the overall energy of the structure.

Figure 6b illustrates the likelihood of the dihedral orientation by way of Boltzmann distribution of the minimized energies. The results show that the preferred orientation for both surfaces is skewed heavily toward the 300° dihedral region, although the (1−10) crystal surface (blue diamonds) has more than a five-fold higher probability compared to the (110) surface (green circles). Notably, the described conformation may be explained in part by hydrogen bonding and proximity of the nearby hydroxyl to an amide bond.

The distance between the transducer surface and the AMC cleavage point as a function of the dihedral was also determined and is plotted in Figure 6c. The blue diamonds present the results for the (1−10) crystal plane calculations. The coumarin cleavage points for the (1−10) models lie between 20 and 30 Å from the transducer surface (mean = 23.0 Å, median = 23.7 Å).

Results from a previous study [27] using a tripeptide–coumarin sensor with a glycine linker instead of PEG found distances for the (1−10) surface of ~10 to 23 Å (mean = 15.1 Å, median = 12.2 Å) between the transducer surface and bond–cleavage point. Note that in the previous study, the cellulose was not TEMPO-oxidized and thus was subject to the *gg*, *tg*, or *gt* conformation of the O6 rotamer, which places further constraints on possible orientations. Nevertheless, the surface–sensor system in the previous study was successfully used as a detector for HNE. Comparing the sensor to transducer surface distances from the rotamer study to the present computational results suggests that HNE would be able to access the cleavage point when using the PEG linker on a TEMPO-oxidized cellulose, as the bond lies further away from the surface, which is supported by the fluorescence activity presented in the previous section.

The results for the (110) surface distance measurements are also provided in Figure 6c (green circles). Compared to the (1−10) surface, the linker and subsequently the bond cleavage point lie much closer to the surface as illustrated in Figure 5b. Because of this, there is a larger amount of non-viable geometries (symbols marked with an “X”), accounting for nearly half of the total calculated structures. On average, the bond–cleavage point is ~9.5 Å from the surface (median = 8.2 Å). Taking into account the possible structures only (no “X”), the mean distance shortens to 6.4 Å, nearly 17 Å closer than on the (1−10) surface and one-third the distance compared to the previous sensor with the glycine linker (17.3 Å) [27]. This is a good illustration of the potential issues for using this PEG linker. Its restricted degrees of freedom, the PEG units preferring the trans conformation and rigid nature of resonance-stabilized amide bonds, prevents the coumarin and its cleavage point from moving freely. For the (1−10) surface, this results in each dihedral orientation accessible to enzyme active site binding at a distance away from the surface. Moreover, these orientations have been judged as accessible by HNE. However, for the (110) surface, the cleavage site is often too close to the surface with the lowest energy conformation, providing only 6 Å between the surface and coumarin bond–cleavage site.

### 2.5. Cotton-Based Nanocellulose Crystallites: A Route to Optimize Predictive Biosensor Conformers

**Figure 7:**
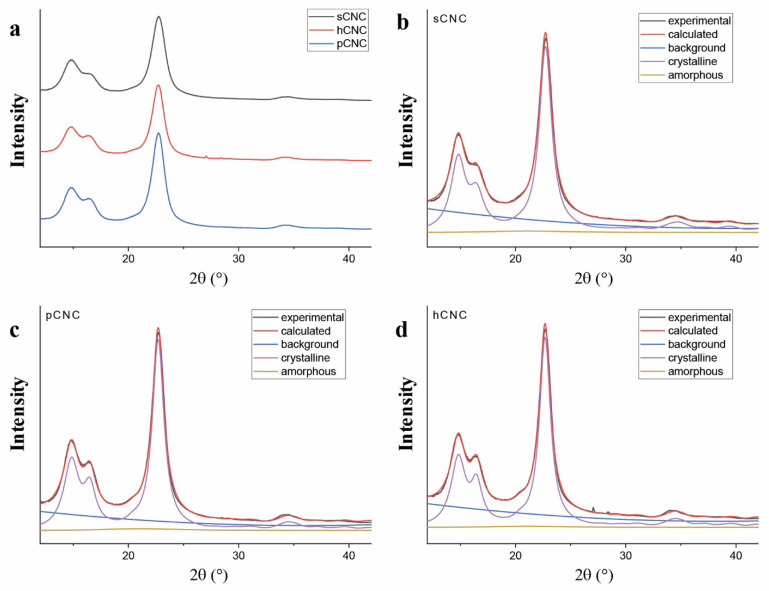
(**a**) Experimental diffraction patterns for lyophilized CNC powder, and (**b**–**d**) results of Rietveld refinement of the entire diffraction pattern for each nanocrystalline material.

The computational models derived in this study suggest that the bioactivity observed with the tNFC-Pep analog of this study is potentially optimizable through adoption of a (1−10) conformation. Selecting materials with a preferred (1−10) crystal plane could potentially increase the number of sensors attached to the transducer surface (sensors per volume) and improve the limit of detection and resolution of the biosensors. Thus, we sought to determine if there is a (1−10) conformer predominantly contained in an accessible crystallite population. Therefore, preliminary X-ray diffraction measurements have been performed on a variety of nanocellulose materials to investigate their predominant crystal plane. Two of the materials contain charged groups on the surface, which have been shown to potentially help control colloidal and thermal stability and environmental interactions, which can alter their typical crystalline structure [51,52,53]. The three materials measured are nanocrystalline cellulose containing sulfate half-esters (sCNC), phosphate half-esters (pCNC), or surface hydroxyls (hCNC). Samples were analyzed using powder X-ray diffraction (XRD). The experimental diffraction patterns are shown in Figure 7. The MAUD Rietveld refinement program was used for analysis of the resulting diffraction patterns [54,55]. The predominant reflections observed for the three CNC derivatives were of the (1–10), (110), and (200) peaks corresponding to the cellulose 1β diffraction pattern located at approximately 14.8°, 16.5°, and 22.8°, 2θ, respectively.

The crystallinity and crystallite size were determined using Equations (1) and (2), respectively, while the *d*-spacings were calculated from the refined unit cell dimensions. The prepared CNCs were crystalline, with crystallinity indexes of 85–90%. The refined unit cell dimensions and *d*-spacings between lattice planes and crystallite sizes were similar in all three instances. In addition, since the (004) reflection is weak and the (012) and (102) reflections are similarly not observed suggests some degree of uniaxial orientation of the rod-like nanocrystals. The March–Dollase approach can be used to quantitatively describe the degree of preferred orientation or distribution of crystallites with respect to a given lattice plane, which are parallel to the sample surface and thus induce the Bragg condition for diffraction. The March parameter *r* defines the degree of preferred orientation, 0 ≤ *r* ≤ 1, where *r* = 0 describes perfect uniaxial orientation and *r* = 1 defines a random powder sample [56].

Extraction of the March parameter *r* using MAUD to apply the Rietveld refinement procedure to the whole diffraction pattern and application of Equation (3) suggests 48–65% orientation of the crystallites, as opposed to a random orientation, with respect to the (001) axis. Further examination of the experimental XRD pattern revealed a significant texture associated with the (1−10) lattice plane. In this instance, the ratio of the intensities between the (1−10) and (110) reflections (κ) was sufficiently large compared to κ_p_, in which Equation (4) was used to extract the March parameter *r*. The results are shown in Table 2, where *η* is the degree of preferred orientation relative to the given *hkl* lattice plane.

The results indicate a strong preferred orientation of the crystallites with respect to the (1−10) lattice plane and alignment of the crystallites during drying. The sCNC sample contains the largest percentage (1−10) crystal plane at 81%, although the other two materials are similarly high at 74%. Additionally, the crystallite sizes for each material are slightly over 5 nm, which is comparable to previously studied cellulose-based materials [20,40]. Similar crystallite sizes but with a prominent (1−10) crystal plane could potentially increase the number of anchored elastase substrates (sensors per volume) and improve the results of the bioactivity assay.

## 3. Materials and Methods

### 3.1. Optical Microscope and FE-SEM of Transducers

Hirox Optical Microscope (Leonia, NJ, USA)equipped with a MGX lens was used to obtain high range images of the transducers with a 350× magnification, a resolution of 0.5 μm, and a scale of 200 μm. The iridescence of the transducers was observed with polarized filters. The samples were placed onto the microscope stage and imaged without further processing.

The field emission scanning electron microscopy (FE-SEM) matrices were imaged using a FEI Quanta 3D FEG FIB/SEM with magnifications of 65,000, 80,000, and 100,000× with a 500 nm scale. The samples were sputter coated with a thin 4 nm layer of gold–palladium using a Leica EM ACE600 (Wetzlar, Germany) sputter coater to ensure that the surface morphology of the matrices were not altered.

### 3.2. Fluorogenic Enzymatic Activity Assay

Stock solutions of the fluorescent substrate were prepared from which serial dilutions were made using a phosphate-buffered saline (PBS) solution consisting of 0.1 M sodium dihydrogen phosphate (NaH_2_PO_4_) and 0.5 M sodium chloride (NaCl) in ultra pure water(Milli-Q, Millipore Sigma, MA, USA). The pH was adjusted to 7.4 with 1 N sodium hydroxide (NaOH) or 1 N hydrochloric acid (HCl) and the PBS buffer filtered with a 0.45 μm filter for all assays.

A standard curve of the substrate was prepared with a serial dilution ranging from 1 to 0.0156 μmol/mL. The biosensor stock solutions were prepared by suspending 20 mg of tNFC-Pep (tNFC with attached bioactive molecule) in 1 mL of PBS, which equals about 2 mg of sample. To start the reaction, 50 μL of human neutrophil elastase at a concentration of 0.06 U/mL was added to the standard curve and to the biosensor to provide a total well volume of 150 μL.

Measurement commenced immediately at 37° C and continued for 1 h at 1 min intervals. The 96-well plate was shaken before each measurement for 3 s. The fluorescent substrate was excited at 360 nm, and the emission was recorded at 460 nm to measure the increase in fluorescence of the amidolytic activity.

### 3.3. Emission Spectroscopy

The emission spectrometry measurements of the 7-amino-4-methylcoumarin (AMC) peptide substrate were obtained using Shimazdu Scientific RF-5301 spectrofluorophotometer (Portland, OR, USA). The substrate solutions 0–0.5 μmol/mL were prepared using the PBS solution and 100 μL were added to the centrifuge tube. A 2 mg sample of biosensor tNFC-Pep was placed into a centrifuge tube with 100 μL of PBS. A volume of 100 μL of the elastase enzyme 1 U/mL was added to the wells containing the substrate and biosensors for a total volume of 200 μL. The centrifuge tube was placed onto an Accu block digital dry bath (Labnet International, Edison, NJ, USA) for 30 min at 37 °C. These samples were diluted (15×) with PBS, 3 mL of each sample were placed in a cuvette, and fluorescence measurements were scanned from 405–625 nm with an excitation at 390 nm.

### 3.4. Computational Modeling and Analysis

The cellulose models (crystallite, (110) crystal plane, and (1−10) crystal plane) were constructed based on cellulose crystallographic files and manipulated with GaussView(Wallingford, CT, USA) molecular building software. The biosensor was constructed in GaussView as well. All energy minimization calculations were performed via Gaussian 16 molecular computational software using the semi-empirical (PM3) method.

The (1−10) and (110) cellulose surfaces were simulated via five adjacent cellulose strands, each containing five glucan units resulting in a total of 25 rings. The C6-hydroxyl was converted to a carboxylic acid for each glucosyl unit where the C6 is on the transducer surface (the face on which the linker is attached). This results in an alternating pattern of oxidized rings due to the inherent 2-fold screw axis of cellulose chains. The PEG linker was attached to the C6 of the central glucan ring, and the attachment conformation was modified for each iterative calculation to find the structure with the lowest overall energy. A semi-empirical method (PM3) was used for the calculations. While a more precise method such as ab initio or DFT could be useful, due to limitations in the computational system, a semi-empirical method allowed for the calculations to finish within a reasonable time frame. Most of the coordinates for the atoms in the cellulose surface were locked to better simulate the rigidity of a crystallite. The only exception was that some hydroxyl groups on the surface were unlocked to understand the role of hydrogen bonding in stabilizing the final structure.

The two surfaces were optimized initially without the biosensor attached to have a baseline geometry for each subsequent calculation. The polyethylene glycol (PEG) linker-fluorogenic elastase substrate was also optimized free of the surface to find its optimal conformation before being attached to the transducer surface. Forty-eight geometries were calculated for each surface, rotating the C2-C5-C6-O_TO_ dihedral at an increment of 7.5°, where each of the carbon atoms are on the same central glucan ring, and the oxygen atom is connected to C6 via a double bond (carbonyl). The optimized energy (in a.u.) was recorded for each calculation and converted into kJ/mol. Not all dihedral angles produced viable structures. Some of the calculations failed outright due to inherent issues with atomic overlap in the starting structures, hence the missing points in the data set for the (110) crystal plane (Figure 5).

The analysis to calculate the distance between the transducer surface and the peptide–coumarin cleavage point was performed in GaussView using the measuring tool and by selecting the nitrogen atom attached to the coumarin and a point on the surface such that the line between them was perpendicular to the surface. For the optimized geometries where the cleavage points were off the side of the surface model, the transducer surface was extended by placing dummy hydrogen atoms to accommodate. In some of the dihedral calculations, the coumarin and other portions of the tripeptide dipped below the surface, causing the overall structure to not be an accurate representation for how the cellulose and linker interact. In reality, the cellulose would extend further out than the model, preventing such a structure. These structures have negative values for the surface–cleavage point distances and are considered non-viable structures for comparison.

### 3.5. Powder X-ray Diffraction

Powder X-ray diffraction (PXRD) measurements were performed on lyophilized samples of cellulose nanocrystals (CNCs) prepared by freeze-drying a 0.5 wt% suspension and examining the obtained solid powder. The CNCs were obtained using mineral acid hydrolysis of isolated cellulose from cotton gin motes [57]. The gin motes were supplied by the USDA (Stoneville, MS). The CNCs examined contained either a hydroxyl moiety (hCNC) or a charged surface group, i.e. sulfate half-esters (sCNC) or phosphate half-esters (pCNC). For XRD analysis, a PANalytical Empyrean laboratory diffractometer (Malvern Panalytical Inc., Westborough, MA, USA) was used with Cu Kα-radiation (1.5406 Å), a spinning, zero-background sample holder at room temperature and a PIXcel3D detector equipped with a 1.0 mm radial divergence slit and a 0.1 mm receiving slit. Powder diffraction patterns were analyzed with the Materials Analysis Using Diffraction (MAUD) Rietveld refinement program (*v*. 2.84) using a pseudo-Voigt peak shape. The crystallographic information files for cellulose Iβ and cellulose II were used for the crystalline and amorphous phases, respectively [54,58]. The isotropic crystallite size for the cellulose Iβ pattern was set to twelve to simulate the amorphous content. The crystallinity index (*CrI*) was calculated using the Rietveld analytical method and was determined from the background subtracted area of the calculated diffraction pattern for crystalline cellulose ($A_{c}$) over the sum of the total contributions from both the crystalline and amorphous ($A_{a}$) phases (Equation (1)) [59].

(1) $$CrI\;\left(\%\right)=\frac{A_{c}}{A_{c}+A_{a}}\times 100\%$$

The Scherrer equation (Equation (2)) was used to calculate the crystallite size, *L* (nm), perpendicular to the (*hkl*) plane [60]:

(2) $$L_{hkl}=\frac{0.9\lambda }{\beta_{hkl}\mathrm{Cos}\theta }$$

where *λ* is the radiation wavelength (1.5406 Å), *θ* is the diffraction angle, and *β_hkl_* is the angular full-width at half maximum height (FWHM), in radians, of the respective line profile. The associated *d*-spacings were calculated from the refined unit cell dimensions.

To calculate the degree of preferred orientation *η*, the March–Dollase approach was used in the analysis of the refined texture and the March parameter *r* extracted from the Rietveld routine in MAUD and applied to Equation (3) [56]:

(3) $$\eta =100\%\;\sqrt[{2}]{\left[\frac{{\left(1-r\right)}^{2}}{(1-r^{3}}\right]}$$

In the case of strong preferred orientation, such as that observed for the reflections observed for the (1−10) lattice plane, the March parameter *r* was determined from Equation (4): [56]

(4) $$r=\sqrt[{3}]{\frac{\sin^{2}\alpha }{\left(\kappa /\kappa_{p}\right)-\cos^{2}\alpha }}$$

where *α* is the angle (in radians) between vectors **H**(*HKL*), and **h**(*hkl*), and where (**H**) is that of the preferred orientation, *κ_p_* is the intensity ratio for a random powder taken from the crystallographic information file, and *κ* is the ratio between the observed intensity of **H** and **h** (i.e., I(**H**)/I(**h**) = *κ*). The March parameter *r* was then used to find *η* by means of Equation (3).

## 4. Conclusions

The results of this study on the tNFC-Pep analog illustrate that TEMPO-oxidized cellulose can be effectively used as a solid support or matrix to immobilize bioactive molecules to detect HNE at levels of 0.5 U/mL, although the fluorescence signal intensity compared to previous materials suggests that the detection of much smaller concentrations is possible. The relatively small crystallite size and mid-range SSA compared to other measured materials results in tNFC having one of the highest numbers of biosensors per unit volume when compared with a wide range of different types of nanocrystalline scaffold motifs. Additionally, tNFC shares properties with a highly porous aerogel-based analog NA-Pep for its substantial swelling when hydrated, thus increasing surface area and access to the enzyme substrate. Therefore, tNFC-Pep can detect more HNE, supported by a higher fluorescence intensity, than the other materials, which implies a lower limit of detection as well. These results suggest that future research should focus on materials that produce smaller crystallites that swell when hydrated, such that there is an increased number of sensors per unit volume, which are readily accessible.

The results of the computational study present a unique opportunity for addressing how to develop biosensors moving forward. By selecting materials that contain crystallites that are predominantly constructed out of the (1−10) crystal plane (i.e., the (1−10) plane is the largest dimension), one could effectively generate a material that has a much higher affinity for peptide binding and for protease access to the enzyme substrate due to fewer steric issues. By increasing the peptide binding and the likelihood of interactions between the sensor and the protease, the material could enhance current devices for POC diagnostic. Preliminary measurements on cellulose materials with charged surface groups suggest that nanocrystalline cellulose with sulfate half-esters on the surface (sCNC) would be a good candidate for further study. The sCNC sample presented the highest amount of (1−10) crystal plane compared to other materials.

Combining the physical properties and theoretical insights could produce novel materials for use as ECM in tissue engineering and wound regeneration in patients with chronic wounds. As an aerogel, tNFC provides a 3D framework, which can be an anchor for peptide-based sensor units. The increased limit of detection and high volume of bioactive molecules in tNFC presents an ideal material to be used as a 3D scaffold for in situ detection of elevated HNE levels in chronic wounds. Modifying the cellulose surface groups could further improve the limit of detection through the selection of crystal planes, which have a preference for biosensor attachment. Substituting different peptide sequences would allow for the detection of other proteases, and combining multiple enzyme substrates on the scaffold could promote detection of a range of issues in patients at once. As such, this study provides the groundwork for designing and developing an array of peptide–cellulose conjugates as a step toward POC diagnostic design in the future.

## Figures and Tables

**Table 1:** Fluorescence intensity of cellulose and nanocellulose materials and corresponding number of sensors per unit volume.

Name	Fluorescence Intensity (3600 s)	Crystallite Volume (nm^3^)	Sensors per Volume	Reference
tNFC-Pep ^a^	320	4.94	8.21	This study
wCNC-Pep	400	7.08	8.78	[20]
wNCC-Pep (66/33) ^b^	80	5.93	3.76	[20]
wNCC-Pep (50/50) ^b^	80	7.08	2.14	[20]
NA-Pep	200	12.84	2.16	[27]
PC-Pep	40	17.94	0.72	[20]
FP-Pep	100	23.7	1.73	[20]

^a^ The abbreviation Pep indicates full bioactive unit (linker, elastase substrate, and fluorophore); ^b^ The NCC materials were composed of a mixture of nanocrystalline and microcrystalline cellulose in the ratio provided after each entry, respectively.

**Table 2:** Results of Rietveld refinement using MAUD for crystallinity index, d-spacings, crystallite sizes, and preferred orientation.

	sCNC	pCNC	hCNC
**Crystallinity (%)**	89.9%	85.8%	90.0%
**d-spacings (Å)**			
(1−10)	5.957	5.946	5.953
(110)	5.361	5.355	5.365
**Crystallite size (nm)**			
(1−10)	5.31	5.60	5.31
(110)	5.55	5.07	4.75
**Preferred orientation (*η*)**			
(001)	48%	65%	61%
(1−10)	81%	74%	74%
(110)	23%	16%	15%

sCNC, sulfate half ester CNCs; pCNC, phosphate half ester CNCs; hCNC, unmodified neutral CNCs.

## Data Availability

Not applicable.
